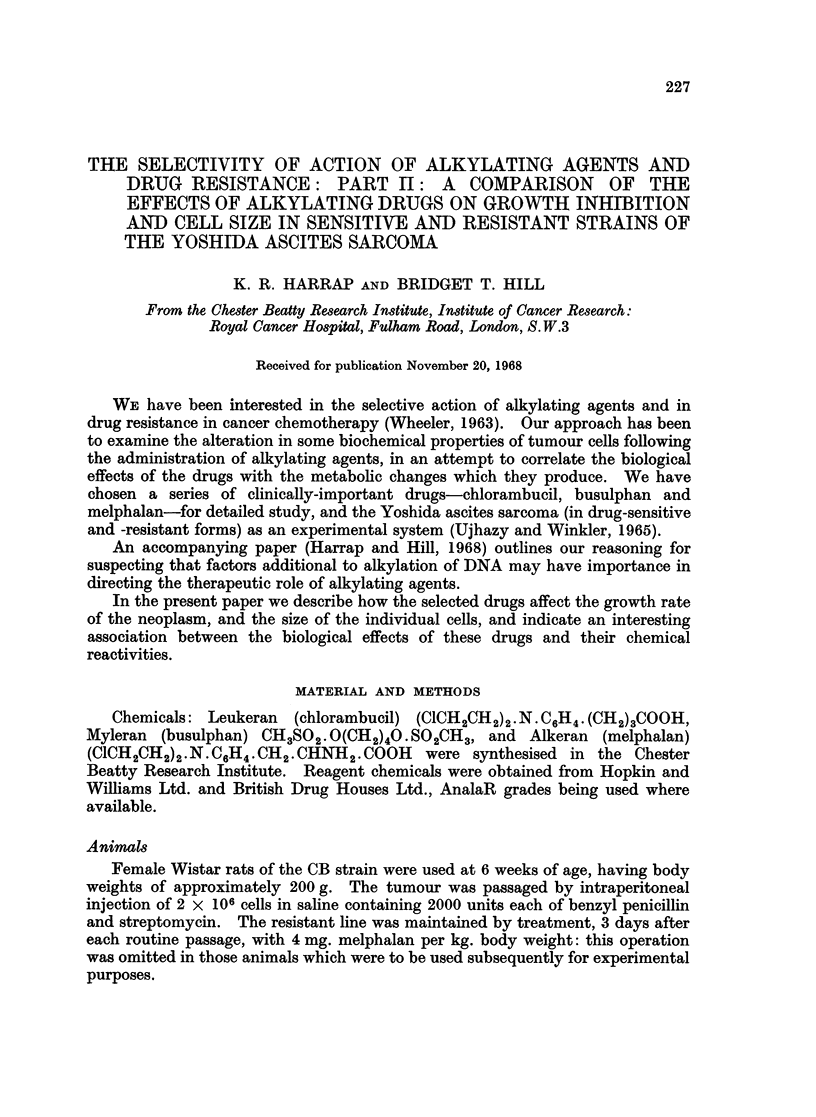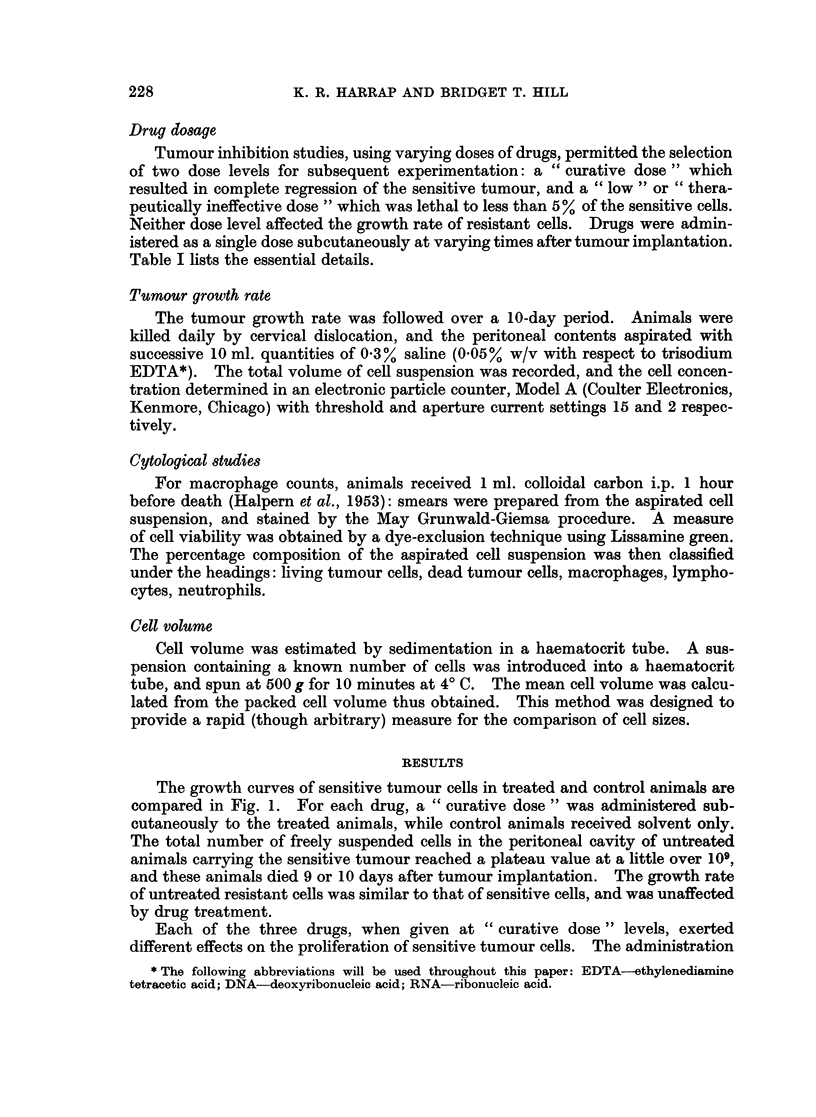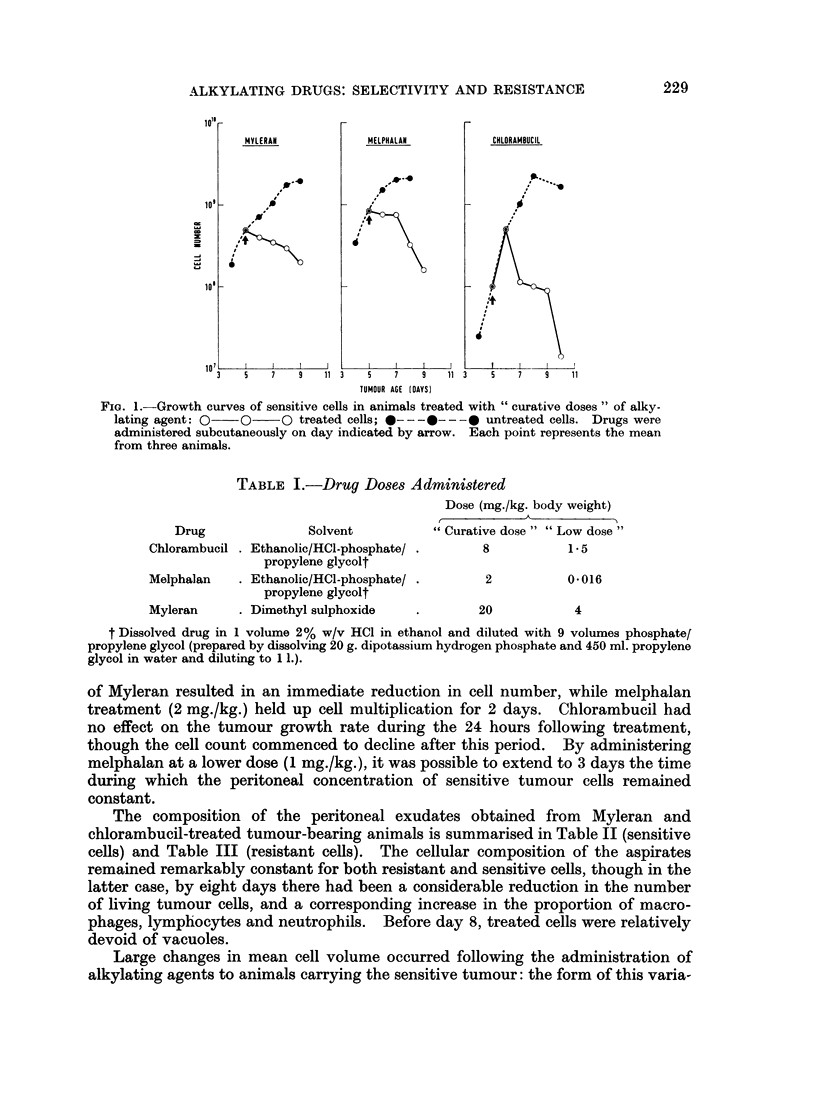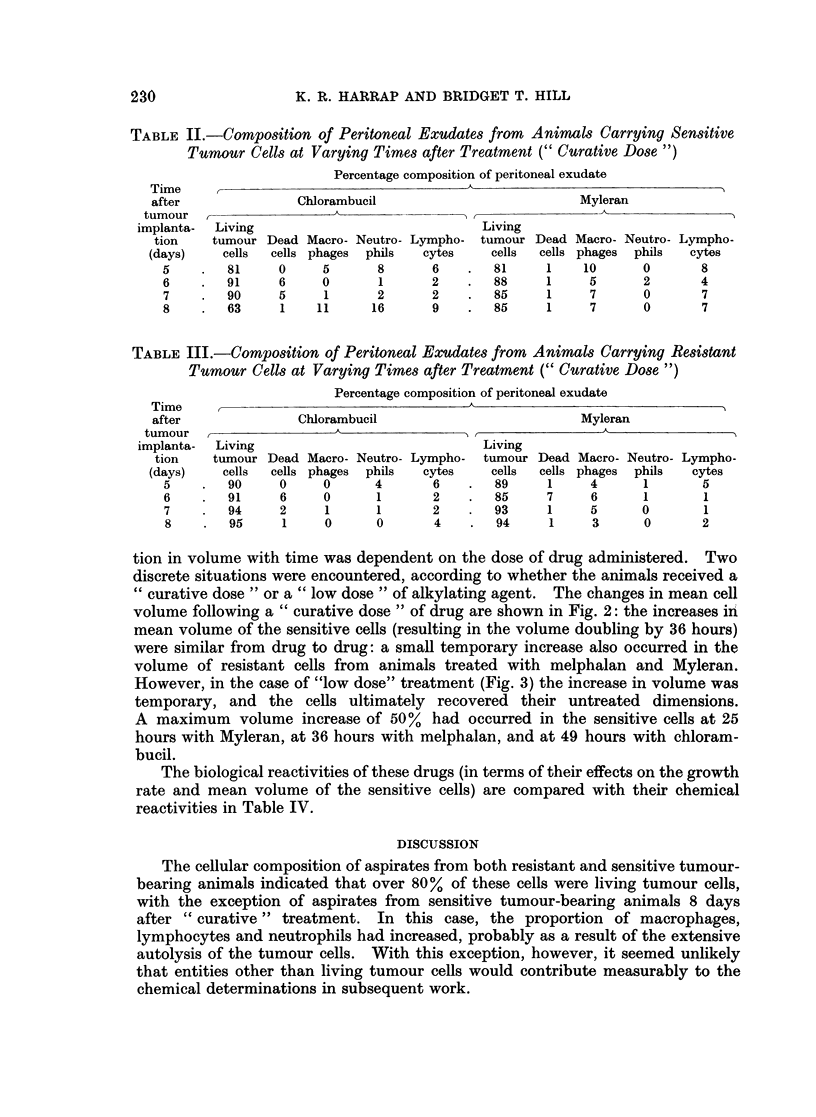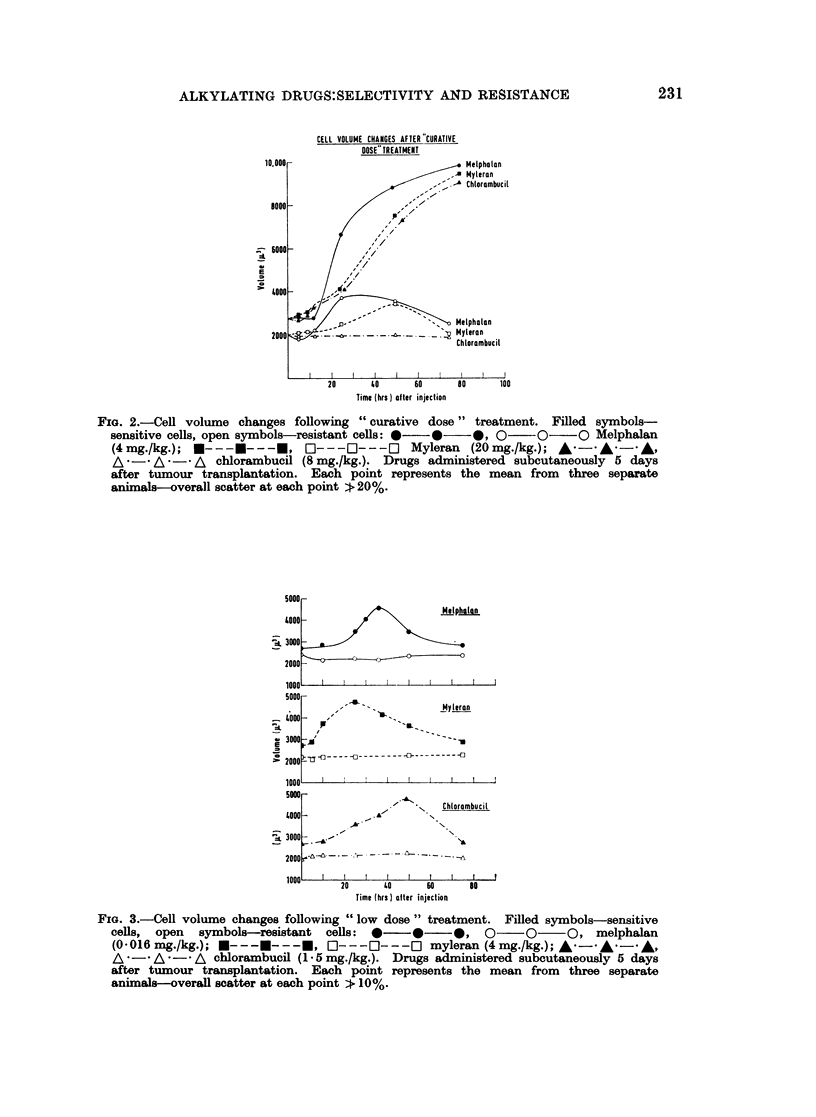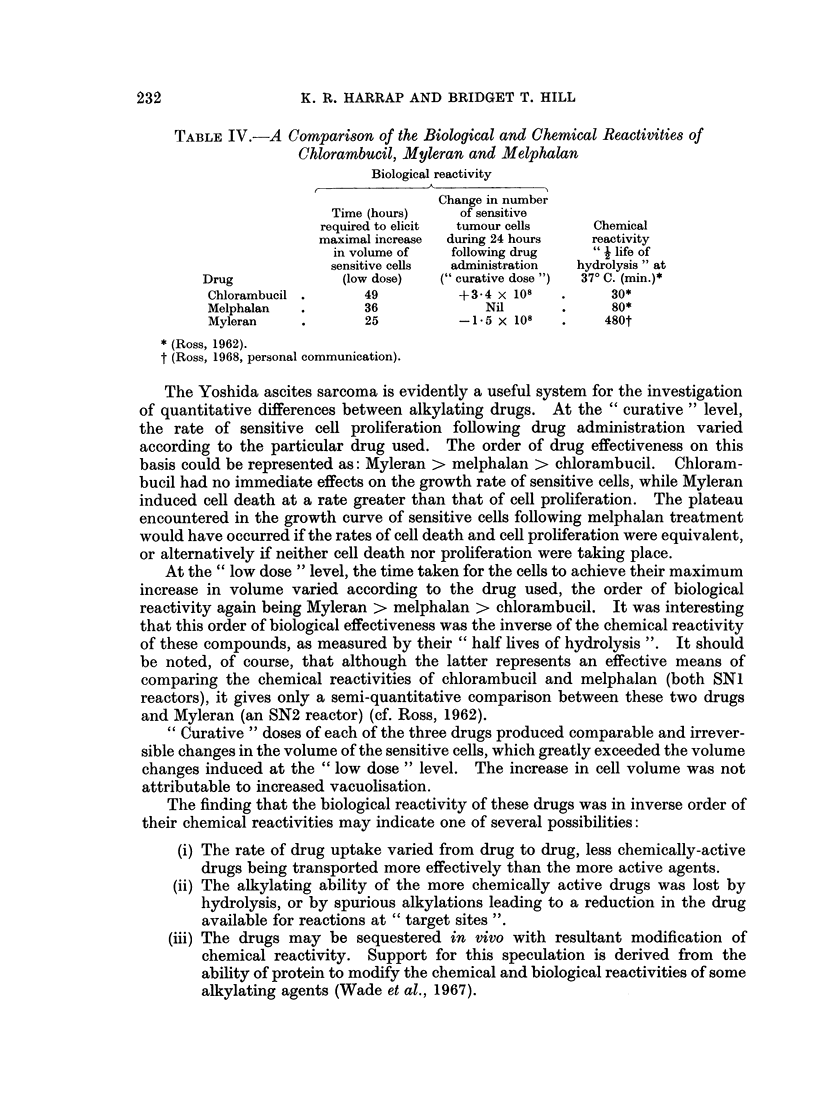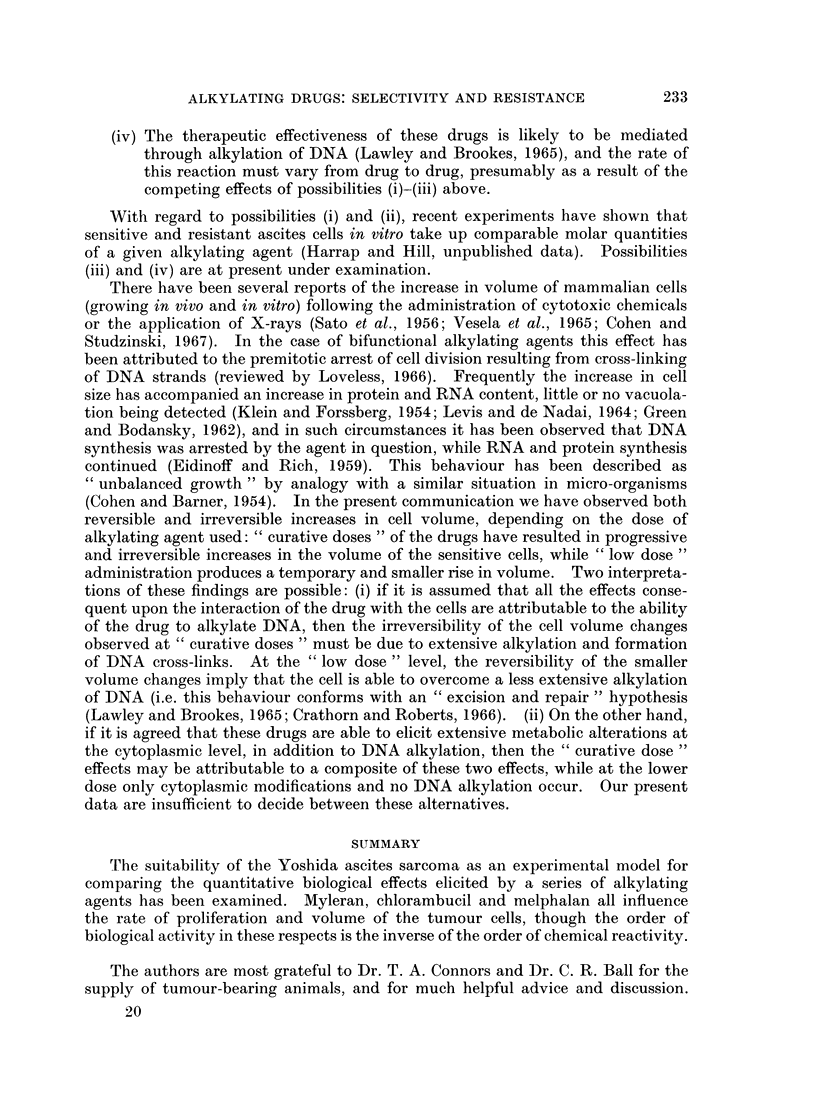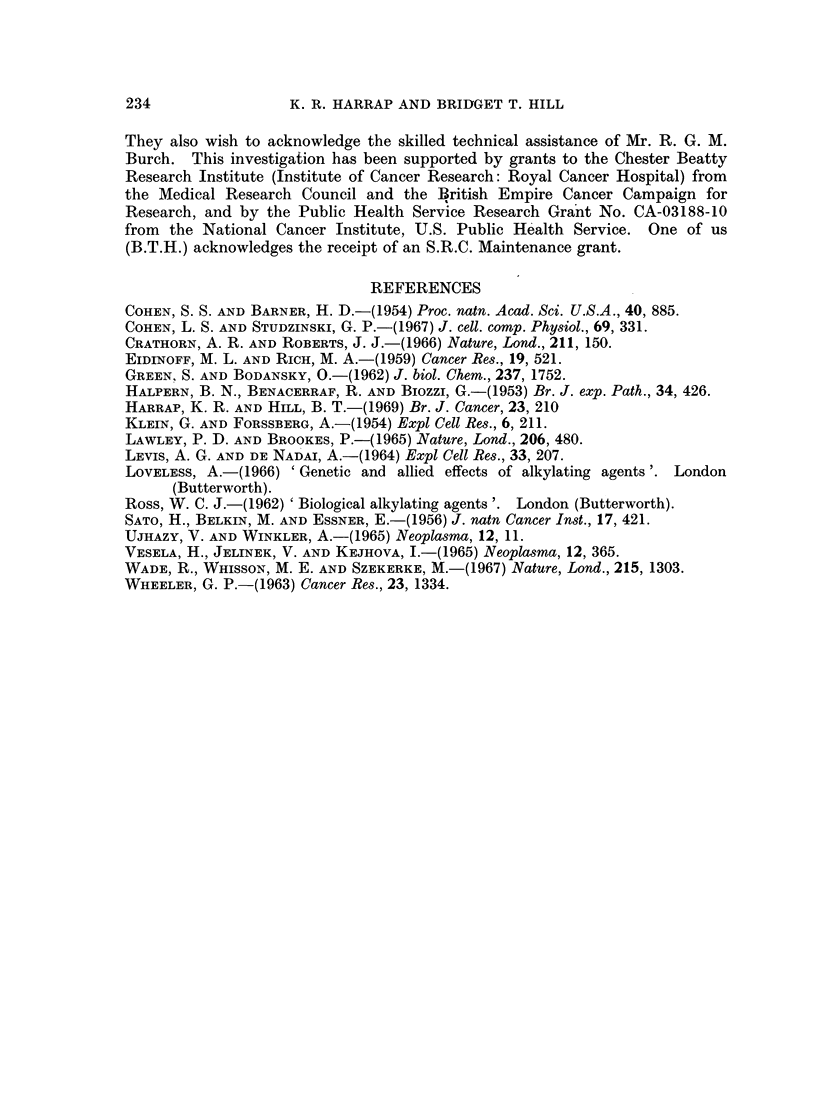# The selectivity of action of alkylating agents and drug resistance. II. A comparison of the effects of alkylating drugs on growth inhibition and cell size in sensitive and resistant strains of the Yoshida ascites sarcoma.

**DOI:** 10.1038/bjc.1969.31

**Published:** 1969-03

**Authors:** K. R. Harrap, B. T. Hill


### THE SELECTIVITY OF ACTION OF ALKYLATING AGENTS AND DRUG RESISTANCE: PART II: A COMPARISON OF THE EFFECTS OF ALKYLATING DRUGS ON GROWTH INHIBITION AND CELL SIZE IN SENSITIVE AND RESISTANT STRAINS OF THE YOSHIDA ASCITES SARCOMA

K. R. HARRAP AND BRIDGET T. HILL

*From the Chester Beatty Research Institute, Institute of Cancer Research: Royal Cancer Hospital, Fulham Road, London, S.W.3*

Received for publication November 20, 1968

WE have been interested in the selective action of alkylating agents and in drug resistance in cancer chemotherapy (Wheeler, 1963). Our approach has been to examine the alteration in some biochemical properties of tumour cells following the administration of alkylating agents, in an attempt to correlate the biological effects of the drugs with the metabolic changes which they produce. We have chosen a series of clinically-important drugs—chlorambucil, busulphan and melphalan—for detailed study, and the Yoshida ascites sarcoma (in drug-sensitive and -resistant forms) as an experimental system (Ujhazy and Winkler, 1965).

An accompanying paper (Harrap and Hill, 1968) outlines our reasoning for suspecting that factors additional to alkylation of DNA may have importance in directing the therapeutic role of alkylating agents.

In the present paper we describe how the selected drugs affect the growth rate of the neoplasm, and the size of the individual cells, and indicate an interesting association between the biological effects of these drugs and their chemical reactivities.

### MATERIAL AND METHODS

Chemicals: Leukeran (chlorambucil) $(ClCH_2CH_2)_2.N.C_6H_4.(CH_2)_3COOH$, Myleran (busulphan) $CH_3SO_2.O(CH_2)_4O.SO_2CH_3$, and Alkeran (melphalan) $(ClCH_2CH_2)_2.N.C_6H_4.CH_2.CHNH_2.COOH$ were synthesised in the Chester Beatty Research Institute. Reagent chemicals were obtained from Hopkin and Williams Ltd. and British Drug Houses Ltd., AnalaR grades being used where available.

### *Animals*

Female Wistar rats of the CB strain were used at 6 weeks of age, having body weights of approximately 200 g. The tumour was passaged by intraperitoneal injection of $2 \times 10^6$ cells in saline containing 2000 units each of benzyl penicillin and streptomycin. The resistant line was maintained by treatment, 3 days after each routine passage, with 4 mg. melphalan per kg. body weight: this operation was omitted in those animals which were to be used subsequently for experimental purposes.

*Drug dosage*

Tumour inhibition studies, using varying doses of drugs, permitted the selection of two dose levels for subsequent experimentation: a " curative dose " which resulted in complete regression of the sensitive tumour, and a " low " or " therapeutically ineffective dose " which was lethal to less than 5% of the sensitive cells. Neither dose level affected the growth rate of resistant cells. Drugs were administered as a single dose subcutaneously at varying times after tumour implantation. Table I lists the essential details.

*Tumour growth rate*

The tumour growth rate was followed over a 10-day period. Animals were killed daily by cervical dislocation, and the peritoneal contents aspirated with successive 10 ml. quantities of 0·3% saline (0·05% w/v with respect to trisodium EDTA*). The total volume of cell suspension was recorded, and the cell concentration determined in an electronic particle counter, Model A (Coulter Electronics, Kenmore, Chicago) with threshold and aperture current settings 15 and 2 respectively.

*Cytological studies*

For macrophage counts, animals received 1 ml. colloidal carbon i.p. 1 hour before death (Halpern *et al.*, 1953): smears were prepared from the aspirated cell suspension, and stained by the May Grunwald-Giemsa procedure. A measure of cell viability was obtained by a dye-exclusion technique using Lissamine green. The percentage composition of the aspirated cell suspension was then classified under the headings: living tumour cells, dead tumour cells, macrophages, lymphocytes, neutrophils.

*Cell volume*

Cell volume was estimated by sedimentation in a haematocrit tube. A suspension containing a known number of cells was introduced into a haematocrit tube, and spun at 500 *g* for 10 minutes at 4° C. The mean cell volume was calculated from the packed cell volume thus obtained. This method was designed to provide a rapid (though arbitrary) measure for the comparison of cell sizes.

### RESULTS

The growth curves of sensitive tumour cells in treated and control animals are compared in Fig. 1. For each drug, a " curative dose " was administered subcutaneously to the treated animals, while control animals received solvent only. The total number of freely suspended cells in the peritoneal cavity of untreated animals carrying the sensitive tumour reached a plateau value at a little over $10^9$, and these animals died 9 or 10 days after tumour implantation. The growth rate of untreated resistant cells was similar to that of sensitive cells, and was unaffected by drug treatment.

Each of the three drugs, when given at " curative dose " levels, exerted different effects on the proliferation of sensitive tumour cells. The administration

* The following abbreviations will be used throughout this paper: EDTA—ethylenediamine tetracetic acid; DNA—deoxyribonucleic acid; RNA—ribonucleic acid.

FIG. 1.—Growth curves of sensitive cells in animals treated with " curative doses " of alkylating agent: ○——○——○ treated cells; ●- - -●- - -● untreated cells. Drugs were administered subcutaneously on day indicated by arrow. Each point represents the mean from three animals.

TABLE I.—*Drug Doses Administered*

| Drug | Solvent | Dose (mg./kg. body weight) " Curative dose " | " Low dose " |
|---|---|---|---|
| Chlorambucil | Ethanolic/HCl-phosphate/ propylene glycol† | 8 | 1·5 |
| Melphalan | Ethanolic/HCl-phosphate/ propylene glycol† | 2 | 0·016 |
| Myleran | Dimethyl sulphoxide | 20 | 4 |

† Dissolved drug in 1 volume 2% w/v HCl in ethanol and diluted with 9 volumes phosphate/ propylene glycol (prepared by dissolving 20 g. dipotassium hydrogen phosphate and 450 ml. propylene glycol in water and diluting to 1 l.).

of Myleran resulted in an immediate reduction in cell number, while melphalan treatment (2 mg./kg.) held up cell multiplication for 2 days. Chlorambucil had no effect on the tumour growth rate during the 24 hours following treatment, though the cell count commenced to decline after this period. By administering melphalan at a lower dose (1 mg./kg.), it was possible to extend to 3 days the time during which the peritoneal concentration of sensitive tumour cells remained constant.

The composition of the peritoneal exudates obtained from Myleran and chlorambucil-treated tumour-bearing animals is summarised in Table II (sensitive cells) and Table III (resistant cells). The cellular composition of the aspirates remained remarkably constant for both resistant and sensitive cells, though in the latter case, by eight days there had been a considerable reduction in the number of living tumour cells, and a corresponding increase in the proportion of macrophages, lymphocytes and neutrophils. Before day 8, treated cells were relatively devoid of vacuoles.

Large changes in mean cell volume occurred following the administration of alkylating agents to animals carrying the sensitive tumour: the form of this varia-

TABLE II.—*Composition of Peritoneal Exudates from Animals Carrying Sensitive Tumour Cells at Varying Times after Treatment (" Curative Dose ")*

| Time after tumour implantation (days) | Percentage composition of peritoneal exudate | | | | | | | | | |
|---|---|---|---|---|---|---|---|---|---|---|
| | Chlorambucil | | | | | Myleran | | | | |
| | Living tumour cells | Dead cells | Macrophages | Neutrophils | Lymphocytes | Living tumour cells | Dead cells | Macrophages | Neutrophils | Lymphocytes |
| 5 | 81 | 0 | 5 | 8 | 6 | 81 | 1 | 10 | 0 | 8 |
| 6 | 91 | 6 | 0 | 1 | 2 | 88 | 1 | 5 | 2 | 4 |
| 7 | 90 | 5 | 1 | 2 | 2 | 85 | 1 | 7 | 0 | 7 |
| 8 | 63 | 1 | 11 | 16 | 9 | 85 | 1 | 7 | 0 | 7 |

TABLE III.—*Composition of Peritoneal Exudates from Animals Carrying Resistant Tumour Cells at Varying Times after Treatment (" Curative Dose ")*

| Time after tumour implantation (days) | Percentage composition of peritoneal exudate | | | | | | | | | |
|---|---|---|---|---|---|---|---|---|---|---|
| | Chlorambucil | | | | | Myleran | | | | |
| | Living tumour cells | Dead cells | Macrophages | Neutrophils | Lymphocytes | Living tumour cells | Dead cells | Macrophages | Neutrophils | Lymphocytes |
| 5 | 90 | 0 | 0 | 4 | 6 | 89 | 1 | 4 | 1 | 5 |
| 6 | 91 | 6 | 0 | 1 | 2 | 85 | 7 | 6 | 1 | 1 |
| 7 | 94 | 2 | 1 | 1 | 2 | 93 | 1 | 5 | 0 | 1 |
| 8 | 95 | 1 | 0 | 0 | 4 | 94 | 1 | 3 | 0 | 2 |

tion in volume with time was dependent on the dose of drug administered. Two discrete situations were encountered, according to whether the animals received a " curative dose " or a " low dose " of alkylating agent. The changes in mean cell volume following a " curative dose " of drug are shown in Fig. 2: the increases in mean volume of the sensitive cells (resulting in the volume doubling by 36 hours) were similar from drug to drug: a small temporary increase also occurred in the volume of resistant cells from animals treated with melphalan and Myleran. However, in the case of "low dose" treatment (Fig. 3) the increase in volume was temporary, and the cells ultimately recovered their untreated dimensions. A maximum volume increase of 50% had occurred in the sensitive cells at 25 hours with Myleran, at 36 hours with melphalan, and at 49 hours with chlorambucil.

The biological reactivities of these drugs (in terms of their effects on the growth rate and mean volume of the sensitive cells) are compared with their chemical reactivities in Table IV.

### DISCUSSION

The cellular composition of aspirates from both resistant and sensitive tumour-bearing animals indicated that over 80% of these cells were living tumour cells, with the exception of aspirates from sensitive tumour-bearing animals 8 days after " curative " treatment. In this case, the proportion of macrophages, lymphocytes and neutrophils had increased, probably as a result of the extensive autolysis of the tumour cells. With this exception, however, it seemed unlikely that entities other than living tumour cells would contribute measurably to the chemical determinations in subsequent work.

**Fig. 2.**—Cell volume changes following "curative dose" treatment. Filled symbols—sensitive cells, open symbols—resistant cells: ●——●——●, ○——○——○ Melphalan (4 mg./kg.); ■- - -■- - -■, □- - -□- - -□ Myleran (20 mg./kg.); ▲·—·▲·—·▲, △·—·△·—·△ chlorambucil (8 mg./kg.). Drugs administered subcutaneously 5 days after tumour transplantation. Each point represents the mean from three separate animals—overall scatter at each point ≯ 20%.

**Fig. 3.**—Cell volume changes following "low dose" treatment. Filled symbols—sensitive cells, open symbols—resistant cells: ●——●——●, ○——○——○, melphalan (0·016 mg./kg.); ■- - -■- - -■, □- - -□- - -□ myleran (4 mg./kg.); ▲·—·▲·—·▲, △·—·△·—·△ chlorambucil (1·5 mg./kg.). Drugs administered subcutaneously 5 days after tumour transplantation. Each point represents the mean from three separate animals—overall scatter at each point ≯ 10%.

TABLE IV.—*A Comparison of the Biological and Chemical Reactivities of Chlorambucil, Myleran and Melphalan*

| | Biological reactivity | | |
|---|---|---|---|
| Drug | Time (hours) required to elicit maximal increase in volume of sensitive cells (low dose) | Change in number of sensitive tumour cells during 24 hours following drug administration (" curative dose ") | Chemical reactivity " ½ life of hydrolysis " at 37° C. (min.)* |
| Chlorambucil | 49 | $+3 \cdot 4 \times 10^8$ | 30* |
| Melphalan | 36 | Nil | 80* |
| Myleran | 25 | $-1 \cdot 5 \times 10^8$ | 480† |

* (Ross, 1962).

† (Ross, 1968, personal communication).

The Yoshida ascites sarcoma is evidently a useful system for the investigation of quantitative differences between alkylating drugs. At the " curative " level, the rate of sensitive cell proliferation following drug administration varied according to the particular drug used. The order of drug effectiveness on this basis could be represented as: Myleran > melphalan > chlorambucil. Chlorambucil had no immediate effects on the growth rate of sensitive cells, while Myleran induced cell death at a rate greater than that of cell proliferation. The plateau encountered in the growth curve of sensitive cells following melphalan treatment would have occurred if the rates of cell death and cell proliferation were equivalent, or alternatively if neither cell death nor proliferation were taking place.

At the " low dose " level, the time taken for the cells to achieve their maximum increase in volume varied according to the drug used, the order of biological reactivity again being Myleran > melphalan > chlorambucil. It was interesting that this order of biological effectiveness was the inverse of the chemical reactivity of these compounds, as measured by their " half lives of hydrolysis ". It should be noted, of course, that although the latter represents an effective means of comparing the chemical reactivities of chlorambucil and melphalan (both SN1 reactors), it gives only a semi-quantitative comparison between these two drugs and Myleran (an SN2 reactor) (cf. Ross, 1962).

" Curative " doses of each of the three drugs produced comparable and irreversible changes in the volume of the sensitive cells, which greatly exceeded the volume changes induced at the " low dose " level. The increase in cell volume was not attributable to increased vacuolisation.

The finding that the biological reactivity of these drugs was in inverse order of their chemical reactivities may indicate one of several possibilities:

(i) The rate of drug uptake varied from drug to drug, less chemically-active drugs being transported more effectively than the more active agents.
(ii) The alkylating ability of the more chemically active drugs was lost by hydrolysis, or by spurious alkylations leading to a reduction in the drug available for reactions at " target sites ".
(iii) The drugs may be sequestered *in vivo* with resultant modification of chemical reactivity. Support for this speculation is derived from the ability of protein to modify the chemical and biological reactivities of some alkylating agents (Wade *et al.*, 1967).

(iv) The therapeutic effectiveness of these drugs is likely to be mediated through alkylation of DNA (Lawley and Brookes, 1965), and the rate of this reaction must vary from drug to drug, presumably as a result of the competing effects of possibilities (i)–(iii) above.

With regard to possibilities (i) and (ii), recent experiments have shown that sensitive and resistant ascites cells *in vitro* take up comparable molar quantities of a given alkylating agent (Harrap and Hill, unpublished data). Possibilities (iii) and (iv) are at present under examination.

There have been several reports of the increase in volume of mammalian cells (growing *in vivo* and *in vitro*) following the administration of cytotoxic chemicals or the application of X-rays (Sato *et al.*, 1956; Vesela *et al.*, 1965; Cohen and Studzinski, 1967). In the case of bifunctional alkylating agents this effect has been attributed to the premitotic arrest of cell division resulting from cross-linking of DNA strands (reviewed by Loveless, 1966). Frequently the increase in cell size has accompanied an increase in protein and RNA content, little or no vacuolation being detected (Klein and Forssberg, 1954; Levis and de Nadai, 1964; Green and Bodansky, 1962), and in such circumstances it has been observed that DNA synthesis was arrested by the agent in question, while RNA and protein synthesis continued (Eidinoff and Rich, 1959). This behaviour has been described as " unbalanced growth " by analogy with a similar situation in micro-organisms (Cohen and Barner, 1954). In the present communication we have observed both reversible and irreversible increases in cell volume, depending on the dose of alkylating agent used: " curative doses " of the drugs have resulted in progressive and irreversible increases in the volume of the sensitive cells, while " low dose " administration produces a temporary and smaller rise in volume. Two interpretations of these findings are possible: (i) if it is assumed that all the effects consequent upon the interaction of the drug with the cells are attributable to the ability of the drug to alkylate DNA, then the irreversibility of the cell volume changes observed at " curative doses " must be due to extensive alkylation and formation of DNA cross-links. At the " low dose " level, the reversibility of the smaller volume changes imply that the cell is able to overcome a less extensive alkylation of DNA (i.e. this behaviour conforms with an " excision and repair " hypothesis (Lawley and Brookes, 1965; Crathorn and Roberts, 1966). (ii) On the other hand, if it is agreed that these drugs are able to elicit extensive metabolic alterations at the cytoplasmic level, in addition to DNA alkylation, then the " curative dose " effects may be attributable to a composite of these two effects, while at the lower dose only cytoplasmic modifications and no DNA alkylation occur. Our present data are insufficient to decide between these alternatives.

### SUMMARY

The suitability of the Yoshida ascites sarcoma as an experimental model for comparing the quantitative biological effects elicited by a series of alkylating agents has been examined. Myleran, chlorambucil and melphalan all influence the rate of proliferation and volume of the tumour cells, though the order of biological activity in these respects is the inverse of the order of chemical reactivity.

The authors are most grateful to Dr. T. A. Connors and Dr. C. R. Ball for the supply of tumour-bearing animals, and for much helpful advice and discussion.

They also wish to acknowledge the skilled technical assistance of Mr. R. G. M. Burch. This investigation has been supported by grants to the Chester Beatty Research Institute (Institute of Cancer Research: Royal Cancer Hospital) from the Medical Research Council and the British Empire Cancer Campaign for Research, and by the Public Health Service Research Grant No. CA-03188-10 from the National Cancer Institute, U.S. Public Health Service. One of us (B.T.H.) acknowledges the receipt of an S.R.C. Maintenance grant.

### REFERENCES

COHEN, S. S. AND BARNER, H. D.—(1954) *Proc. natn. Acad. Sci. U.S.A.*, **40**, 885.
COHEN, L. S. AND STUDZINSKI, G. P.—(1967) *J. cell. comp. Physiol.*, **69**, 331.
CRATHORN, A. R. AND ROBERTS, J. J.—(1966) *Nature, Lond.*, **211**, 150.
EIDINOFF, M. L. AND RICH, M. A.—(1959) *Cancer Res.*, **19**, 521.
GREEN, S. AND BODANSKY, O.—(1962) *J. biol. Chem.*, **237**, 1752.
HALPERN, B. N., BENACERRAF, R. AND BIOZZI, G.—(1953) *Br. J. exp. Path.*, **34**, 426.
HARRAP, K. R. AND HILL, B. T.—(1969) *Br. J. Cancer*, **23**, 210
KLEIN, G. AND FORSSBERG, A.—(1954) *Expl Cell Res.*, **6**, 211.
LAWLEY, P. D. AND BROOKES, P.—(1965) *Nature, Lond.*, **206**, 480.
LEVIS, A. G. AND DE NADAI, A.—(1964) *Expl Cell Res.*, **33**, 207.
LOVELESS, A.—(1966) ' Genetic and allied effects of alkylating agents '. London (Butterworth).
ROSS, W. C. J.—(1962) ' Biological alkylating agents '. London (Butterworth).
SATO, H., BELKIN, M. AND ESSNER, E.—(1956) *J. natn Cancer Inst.*, **17**, 421.
UJHAZY, V. AND WINKLER, A.—(1965) *Neoplasma*, **12**, 11.
VESELA, H., JELINEK, V. AND KEJHOVA, I.—(1965) *Neoplasma*, **12**, 365.
WADE, R., WHISSON, M. E. AND SZEKERKE, M.—(1967) *Nature, Lond.*, **215**, 1303.
WHEELER, G. P.—(1963) *Cancer Res.*, **23**, 1334.